# Pontine Tegmental Cap Dysplasia: A Rare Case in South Africa

**DOI:** 10.7759/cureus.96597

**Published:** 2025-11-11

**Authors:** Thobeka Nyila, Gopolang Mndebele, Nonceba Koranteng, Gary Peiser

**Affiliations:** 1 Diagnostic Radiology, Nelson Mandela Children's Hospital, Johannesburg, ZAF; 2 Diagnostic Radiology, University of the Witwatersrand, Johannesburg, Johannesburg, ZAF

**Keywords:** brainstem, congenital, cranial nerves, pontine tegmental cap dysplasia, temporal bones

## Abstract

We present the first case of pontine tegmental cap dysplasia (PTCD) reported in South Africa, involving a one-year-old girl with bilateral sensorineural deafness, global atrophy, hemivertebrae, and butterfly vertebrae. Clinical examination revealed subtle dysmorphic features, abnormal corneal sensation, bilateral strabismus, and uncertain visual acuity.

Magnetic resonance imaging (MRI) demonstrated key diagnostic features, including ventral pons hypoplasia, ectopic dorsal pontine tissue, and cranial nerve abnormalities such as hypoplasia of the facial nerves, absent right vestibulocochlear nerve, and non-branching left vestibulocochlear nerve.

A review of the literature highlights the importance of advanced neuroimaging techniques, such as MRI and diffusion tensor imaging (DTI), in identifying the structural aberrations and potential axonal guidance abnormalities associated with PTCD. This case report aims to shed light on this rare condition's intricacies and emphasize the need for further research to deepen our understanding and enhance patient care outcomes.

## Introduction

Pontine tegmental cap dysplasia (PTCD) is a rare neurological condition that presents many challenges, characterized by hindbrain malformations, cranial nerve deficits, and a spectrum of associated symptoms such as hearing loss and speech impairments [1-3]. Through advanced neuroimaging techniques, including magnetic resonance imaging (MRI), clinicians can delve deeper into the structural aberrations within the pontine region, unveiling key diagnostic features such as ventral pons hypoplasia and ectopic dorsal pontine tissue [2,3]. Although the etiology remains elusive, genetic mutations and aberrant axonal guidance pathways may underlie the pathogenesis of PTCD [4]. This article presents, to the best of our knowledge, the first reported case of PTCD in South Africa and the entire African continent. By reviewing existing literature on the clinical features and diagnostic challenges of PTCD, this case report aims to offer important insights into the complexities of this rare condition.

## Case presentation

We present a one-year-old girl who was referred to our institution for MRI of the brain, with bilateral sensorineural deafness, and was being worked up for cochlear implantation. The patient was reported to have global brain atrophy on the previous MRI of the brain. She was also noted to have hemivertebrae and butterfly vertebrae of T5 to T12.

On clinical examination, the patient had subtle dysmorphic features: epicanthic fold and low-set ears, low central tone with increased peripheral tone, and increased reflexes. She had reduced corneal sensation, bilateral strabismus, and poor visual acuity.

Microarray comparative genomic hybridization (CGH) was found to be normal.

MRI was done to assess the brain, cranial nerves, and internal auditory meati and inner ear structures. Sagittal 3D T1-weighted sequences with reformats, axial and coronal T2-weighted, axial T2-weighted fluid-attenuated inversion recovery (FLAIR), diffusion-weighted imaging (DWI), apparent diffusion coefficient (ADC), and gradient echo (GRE) sequences were performed through the brain. Axial CUBE T2-weighted and axial 3D T2-weighted sequences were performed through the internal acoustic meati.

**Figure 1:**
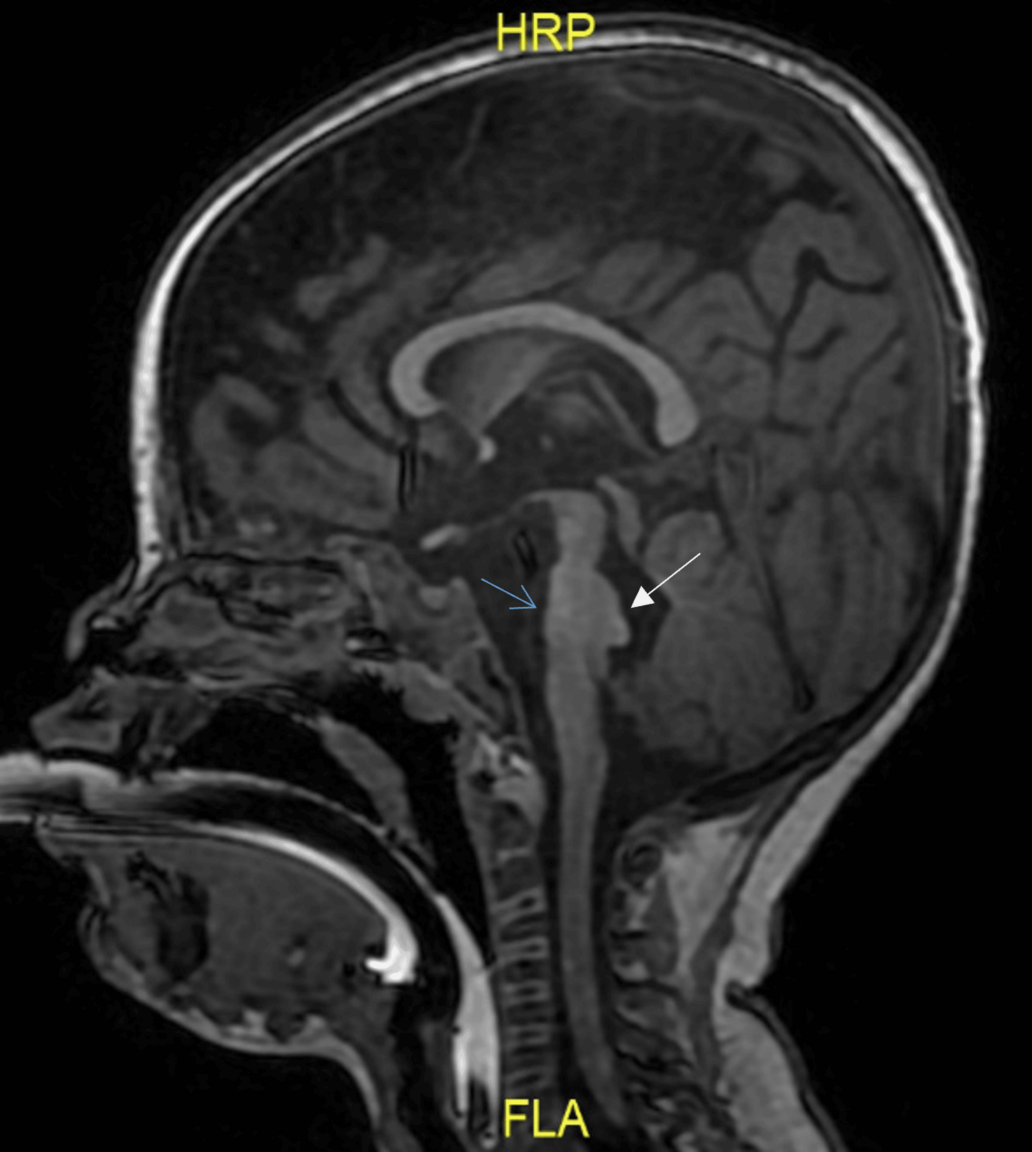
Sagittal T1-weighted image shows hypoplastic pons with flattening of the ventral surface (blue arrow) and dorsal projecting white matter cap (white arrow) into the fourth ventricle.

**Figure 2:**
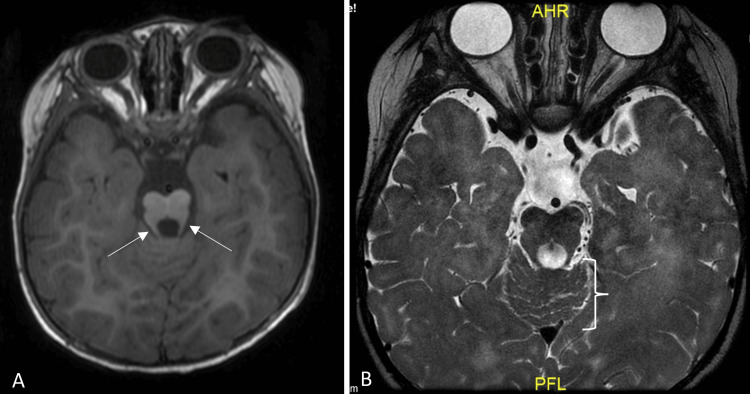
A: Mild lateral bulging and elongation of the superior cerebellar peduncles at the level of the pontomesencephalic junction, demonstrating a molar tooth configuration (arrows). B: Disorganization and anatomical distortion of the vermian lobules (bracket). A: Axial T1-weighted image of the brain at the level of the pons and vermis. B: Axial T2-weighted image of the brain at the level of the pons and vermis.

MRI of the brain demonstrated flattening of the ventral pons with a dorsal projecting white matter cap, associated molar tooth configuration at the pontomesencephalic junction, and dysplastic vermis lobule (Figure 1 and Figure 2). Unfortunately, diffusion tensor imaging (DTI) was not performed.

**Figure 3:**
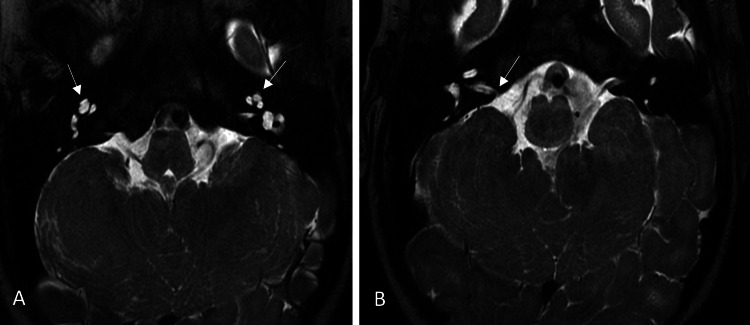
A: Isolated cochleae bilaterally with the absence of the cochlear canals (arrows). B: Narrowing of the right internal acoustic meatus with stenosis at the porus acusticus internus (arrow). A and B: Axial T2-weighted high-resolution images of the internal acoustic meati and inner ears.

The external auditory canals were unremarkable. The T2-weighted high-resolution axial and sagittal planes demonstrated abnormal cranial nerves with hypoplasia of the facial nerves, absent right vestibulocochlear nerve, and non-branching left vestibulocochlear nerve. The cochleae still demonstrated normal configuration, although they were isolated (Figure 3). The vestibules, vestibular aqueducts, and semicircular canals were normal in size and morphology.

## Discussion

To the best of our knowledge, there have been 60 reported cases of PTCD in the literature [1-15], with the first reported case in 2007 by Barth et al. [1]. Additionally, the first description of the key brainstem findings in PTCD was reported by Maeoka et al. in 1997, although the diagnosis of PTCD was not made [16]. A further case, reported by Ouanounou et al. in 2005, described the same brainstem features as PTCD with internal auditory canal stenosis [17]. However, the case was diagnosed as Möbius syndrome with unusual findings [17]. To date, no reported cases have come out of South Africa or Africa as a whole.

Reported clinical findings of PTCD include ataxia, sensorineural hearing loss, speech disturbances, facial palsy, spine anomalies, swallowing problems, central sleep apnea, and failure to thrive [7,8,10].

Advanced radiological imaging, such as MRI with diffusion tensor imaging (DTI) sequence, plays a key role in identifying specific abnormalities characteristic of PTCD. MRI scans reveal critical features such as ventral pons hypoplasia and ectopic dorsal pontine tissue, aiding in the accurate identification of this hindbrain malformation [1-3,5,8]. Additionally, DTI investigations provide insights into potential axonal guidance abnormalities and neuronal migration [2].

Studies have highlighted the significance of differentiating PTCD from related syndromes like Joubert syndrome based on distinct radiological characteristics, where they both have a molar tooth configuration of the superior cerebellar peduncles [1,4].

Individuals with PTCD frequently show cranial nerve impairments, such as hearing loss and speech difficulties. Reported imaging findings of cranial nerve anomalies manifest with bilateral absent cochlear nerves, absent facial nerve, absent vestibulocochlear nerve, small-caliber facial nerve and vestibulocochlear nerve, and optic nerve atrophy [1,4,5,7,9,12]. Temporal bone and inner ear abnormalities include internal auditory canal stenosis, duplicated internal auditory canals, bilateral isolated cochlea, and hypoplastic cochlear nerve canal [1,4-6,9,13-15].

Table 1 provides key imaging findings and variable findings associated with PTCD.

**Table 1:** List of key imaging findings and variable findings associated with PTCD. PTCD: pontine tegmental cap dysplasia

Key imaging findings that were constant across reported cases [1-12]	Other variable reported findings [6,10-12]
Flattening of the ventral pons with a dorsal projecting tegmental cap into the fourth ventricle	Hypoplastic orbit, which was associated with optic nerve atrophy
Molar tooth configuration at the pontomesencephalic junction	Vertebral anomalies: butterfly vertebrae and hemivertebrae
Dysplastic or hypoplastic vermis	Cardiovascular anomalies: ventricular septal defect, atrial septal defect, patent ductus arteriosus, aortic arch hypoplasia/interrupted aortic arch, and *tetralogy*of Fallot
Absent or hypoplastic middle cerebellar peduncles	
Aberrant transverse fiber bundle at the pontine tegmentum on diffusion tensor imaging	

Our case revealed similar key brainstem findings, with cranial nerve, temporal bone, and vertebral anomalies. The radiological findings described above directly correlate with the patient's symptoms.

Evidence from the literature implies a genetic foundation for this syndrome, which may result in fewer nerve fibers and misaligned neural pathways, ultimately causing cranial nerve dysfunction [6,11]. However, no definitive genetic mutations responsible for this condition have been identified.

## Conclusions

Through an in-depth analysis of existing literature and case reports, several key findings have emerged regarding PTCD. Notably, it is characterized by hindbrain malformations, cranial nerve deficits, and developmental delays, which often affect speech and hearing abilities. Radiological investigations play a pivotal role in diagnosing PTCD, with MRI and DTI sequences revealing specific features such as ventral pons hypoplasia and abnormalities in the pontine tegmentum. Genetic etiology remains elusive; therefore, understanding the unique radiological characteristics of PTCD is crucial for accurate diagnosis, prognostic assessment, and tailored treatment strategies. Ongoing research in this field, especially the genetic component, is warranted to deepen our knowledge of this condition and to enhance patient care outcomes.
